# Using neural networks and evolutionary information in decoy discrimination for protein tertiary structure prediction

**DOI:** 10.1186/1471-2105-9-94

**Published:** 2008-02-11

**Authors:** Ching-Wai Tan, David T Jones

**Affiliations:** 1 Department of Computer Science, University College London, London, UK

## Abstract

**Background:**

We present a novel method of protein fold decoy discrimination using machine learning, more specifically using neural networks. Here, decoy discrimination is represented as a machine learning problem, where neural networks are used to learn the native-like features of protein structures using a set of positive and negative training examples. A set of native protein structures provides the positive training examples, while negative training examples are simulated decoy structures obtained by reversing the sequences of native structures. Various features are extracted from the training dataset of positive and negative examples and used as inputs to the neural networks.

**Results:**

Results have shown that the best performing neural network is the one that uses input information comprising of PSI-BLAST [1] profiles of residue pairs, pairwise distance and the relative solvent accessibilities of the residues. This neural network is the best among all methods tested in discriminating the native structure from a set of decoys for all decoy datasets tested.

**Conclusion:**

This method is demonstrated to be viable, and furthermore evolutionary information is successfully used in the neural networks to improve decoy discrimination.

## Background

In recent years, a rise in the number of genome sequencing projects around the world has led to an increase in the number of protein sequences with unknown structures. Protein structure prediction aims to bridge the gap between the number of such sequences and the number of sequences with experimentally determined structures. One advantage of computational protein structure prediction is that accurate *in silico *protein modelling can help guide the more expensive experimental efforts in protein structure determination. Ultimately, the goal is to understand protein function through its 3D structure and sequence and to further increase our biological insights of the behaviour and interactions of these macromolecules in ways that would be beneficial to mankind.

Since 1994, the CASP experiments [2] have provided a useful platform for structure prediction groups to apply their methods to a common set of target sequences, thereby providing the means of direct comparison between these methods. If a target sequence has templates in the structure databases, comparative modelling and fold recognition methods are used to select the templates. In the event of a sequence having an unknown fold, in the case of the Template Free category, fragment assembly methods such as FRAGFOLD [3] and Rosetta [4] are used to build plausible models. Typically, large numbers of candidate models, also known as decoys, are built in order to sample as large a 3D conformational space as possible. The subsequent challenge is to select the lowest RMSD structure among these decoys to represent as the prediction. This is referred to as the decoy discrimination problem. Common approaches to decoy discrimination include the use of statistical potentials [5,6], structural clustering of decoy structures [7,8], as well as the application of Model Quality Assessment Programs (MQAPs) such as MODCHECK [9] and Victor/FRST [10].

In this work, we present a novel method of decoy discrimination using machine learning, more specifically using neural networks. Decoy discrimination is represented as a machine learning problem, where neural networks are used to learn the native-like features of protein structures using a set of positive and negative training examples. A set of native protein structures forms the positive training examples, while a set of simulated decoy structures makes up the negative training examples. Various features are extracted from the training dataset of positive and negative examples and used as inputs to the neural networks.

For the purpose of providing negative training examples, the method of generating a decoy structure (as a negative example) by retaining the physical structure and changing only the sequence ensures that we are using good structural features, as opposed to having overly compact generated structures with steric clashes or non-compact generated structures with obvious non-protein like features. Each of these generated structures with reversed sequences retains native-like structural features, such as native shape and packing density but sequence-related features such as the distributions of pairwise distances of particular residue pairs would be different. For example, the distribution of Alanine-Alanine pairs in native structures 6 residues apart would have a peak at about 11Å typical of helices, but no such peak exists in the distribution in generated structures with reversed sequences. It is hoped that neural network training can be used to capture the differences in these distributions of pairwise distances among the 400 possible residue pair types, and that the final neural network can be used to judge or discriminate decoys with near-native features from poorer quality decoys.

It is also an option to generate near-native decoy structures from native structures and then use the generated structures as negative training examples. In this case, structural features such as the pairwise distances would be different, and the distributions of pairwise distances from such decoy structures would be dissimilar to those of native structures too. In this paper, we have decided to leave this method for future work, and concentrate solely on the sequence reversal method for the generation of negative training examples, and test this sequence reversal method to see if it is effective in the context of neural networks. The other main alternative would be to use decoy datasets as negative training examples, but that would render the decoy datasets unavailable for testing, and most publicly available decoy sets are very limited e.g. in terms of protein sizes or types.

Although machine learning has been used for model quality assessment before [11-13], it has generally used to optimally combine component features such as solvation energy and secondary structure similarity into a single model quality score. In this case we use machine learning to learn features which are the equivalent of basic pairwise and solvation potential terms to discriminate native folds from decoys. The intended advantage of this approach is that a wide range of input features can be directly incorporated into a single scoring function and combined implicitly. For example, close interactions between positively charged and negatively charged amino acid side chains are rare when the side chains are buried. Nevertheless such salt-bridge interactions do occur, and these can be highly discriminatory features when present in the native structure. The rarity of these features, however, makes it hard for traditional statistical pair potential functions to correctly recognize them. If the evolutionary conservation of the charged amino acids is taken into account, then it is straightforward to identify the rare occasions on which a salt-bridge should be considered, as buried salt-bridges are generally seen to be highly conserved across a family of related proteins.

In this work we start out by using neural networks to replace standard pair and solvation potentials. This novel paradigm of using neural networks to perform decoy discrimination is then extended to include evolutionary information. The benefits of using PSI-BLAST [1] profiles with neural networks has previously been demonstrated for secondary structure prediction (e.g. in PSIPRED [14]) and here we demonstrate the use of such information for tertiary structure prediction. Dong and co-workers [15] have also tried to use profile information to develop a better mean force potential for discriminating native structures from sets of decoys, but in their case they simply threshold the sequence profile scores to render a binary decision as to whether a residue is conserved or not conserved. In this paper, we demonstrate that by using neural networks to represent the scoring function, evolutionary information can be encoded as continuous input features to improve the decoy discrimination process. Our intention is to demonstrate the promise of using neural networks with evolutionary information to perform effective coarse decoy discrimination as an initial step, and to provide a platform for future higher resolution decoy discrimination. To provide a rigorous test of these neural network methods, high resolution decoy datasets such as the Baker dataset [16], as well as lower resolution decoy datasets, are used for the comparison of results to that obtained from existing in-house pairwise potential methods [17].

## Results

A set of 475 protein domains are selected from the SCOP database [18] (see Methods, Dataset of Protein domains for more details) and divided into training, validation and preliminary test datasets, of size 60%, 20% and 20% respectively. The number of protein domains in each dataset and the percentages of secondary structure content are shown in Table 1. The training and validation datasets are used for neural network training. The preliminary test dataset is used as a basic test for the neural networks in their ability to distinguish native structures from random decoys. Random decoys are generated by randomizing the sequence of each structure in the preliminary test dataset.

**Table 1:** Number of proteins in the training, validation and preliminary test datasets.

**Dataset**	**Number of proteins**			
	
	**All**	**α-only**	**β-only**	**αβ**
Training	285	58 (20.4%)	59 (20.7%)	168 (58.9%)
Validation	95	18 (19.0%)	25 (26.3%)	52 (54.7%)
Preliminary Test	95	22 (23.2%)	17 (17.9%)	56 (58.9%)

While random decoys provide a basic test, ultimately the neural networks need to be tested on the discrimination of near-native decoys in real decoy datasets. Several decoy datasets are used, and these are shown in Table 2. In the discussion of results, the Baker dataset [16] is discussed in greater detail because it has the highest quality of decoys, compared to other datasets (see Methods, Decoy datasets for testing for more details).

**Table 2:** Decoy datasets used for testing

**Decoy Dataset**	**Number of proteins**
Baker (Tsai) decoy	22
4state_reduced	6
lattice_ssfit	8
fisa	4
fisa_casp3	4
lmds	10
lmds_v2	10
semfold	6

Several input features of the training datasets are extracted and used for neural network training (see Methods, Neural network input features for more details). The various types of neural networks and their input features are shown in Table 3 (non-evolutionary features). For example, the NN-dist method trains neural networks that code for residue pair identities and pairwise distances, as well as having one neural network for each sequence separation *k*. Due to the large size of training data, each discrete value of the sequence separation *k *has to be represented as one neural network. Such a representation means that the various sequence separations *k *have to be combined in meaningful ways. In this work, for the sake of comparison with the in-house pairwise potentials method [17] which has proven competitive in recent CASP experiments, 3 methods of combinations of results are attempted. These are the short-range (S) combination which sums up the average scores from the *k *= 4 to *k *= 10 networks; the short-and-medium (SM) combination which sums up the average scores from the *k *= 4 to *k *= 22 networks; the short-medium-long (SML) combination which sums up the average scores from the *k *= 4 to *k *= 22, as well as *k *> 22, networks (See Methods, Interpretation of network output for more details).

**Table 3:** Summary of neural network training paradigms

**Name**	**Input Features**	**No. of networks**	**Network input size**
NN-dist	Residue pair identities, and pairwise distance	20	41
NN-solvpair	Residue pair identities, and relative solvent accessibilities	20	42
NN-solvpairndist	Residue pair identities, pairwise distance and relative solvent accessibilities	20	43

Besides using the in-house pairwise potentials method [17] as a basis for effective comparison, the K Nearest Neighbours method, a basic machine learning method, is also used for comparison to the various types of neural networks. The measures used for evaluating the different methods are the Z-score and the enrichment score [16]. The Z score measures the extent to which a method can select the native structure from among the decoys; the enrichment score [16] is an indication of the degree to which the method succeeds in identifying the lowest RMSD near-native structures.

The ability of any two decoy discrimination methods can be compared by looking at the difference in the quality of the top-ranked models produced by each method [9]. Here, 3 structural similarity measures for assessing the quality of a decoy model are used, namely TM-score [19], GDT-TS [20] and MaxSub [21]. The one-tailed Wilcoxon sign-rank test is used to test, at a 5% significance level, the differences between any two methods. The null hypothesis is the zero median of the distribution of differences in a given structural similarity score, e.g. TM-score [19], of the respective top-ranked models. Similarly, apart from looking only at the top-ranked model, the ability of any two methods to effectively rank the decoys from highest to lowest quality are also assessed in the form of the Spearman rank correlation coefficient. Here another one-tailed Wilcoxon sign-rank test is used to test the difference between any two methods in their ability to rank the decoys from highest to lowest quality. The null hypothesis is the zero median of the distribution of differences in Spearman correlation coefficients produced by the two methods.

Results produced from neural networks which use evolutionary features extracted from the training datasets are also discussed. Table 4 shows the various types of neural networks that use evolutionary features. A complete description of each method can be found in the section Methods, Inclusion of evolutionary information.

**Table 4:** Summary of methods used for inclusion of evolutionary information

**Variant Type**	**Previous network used**	**No. of input neurons**	**Name for this variant**	**Training required**
Homologue Threading	NN-dist	41	HT-NN-dist	No
Homologue Threading	NN-solvpair	42	HT-NN-solvpair	No
Homologue Threading	NN-solvpairndist	43	HT-NN-solvpairndist	No
Sequence Profile	None	41	SP-NN-dist	Yes
Sequence Profile	None	42	SP-NN-solvpair	Yes
Sequence Profile	None	43	SP-NN-solvpairndist	Yes

### Preliminary test dataset

For each of the 95 proteins in the preliminary test dataset in Table 5, 50 random decoy structures are generated by shuffling the sequence in the native structure.

**Table 5:** Preliminary test dataset of 95 proteins

**Preliminary Test Dataset : Protein {:Chain}{:Domain Boundaries}**			
1eye:A	1ks2:A:127–198	1bm8	1kjq:A:319–392
1fcy:A	1lm5:A	1d3v:A	1kwn:A
1fkm:A:249–442	1lpl:A	1dk8:A	1l0i:A
1g8e:A	1ls1:A:1–88	1dmg:A	1l3l:A:2–169
1gci	1m5w:A	1doz:A	1luc:A
1gk8:A:150–475	1m9x:C	1fma:E	1lyv:A
1gwu:A	1mmg:34–79	1fpo:A:1–76	1m22:A
1h8e:A:380–510	1moq	1g87:A:457–614	1m4j:A
1hdh:A	1mwp:A	1gte:A:2–183	1mky:A:359–439
1hxn	1n08:A	1gxj:A	1mro:A:270–549
1i1q:A	1n63:C:178–287	1h2w:A:1–430	1mzg:A
1j98:A	1nox	1h7m:A	1o7n:A:155–448
1jbe:A	1nz0:A	1hty:A:412–522	1oac:A:5–90
1jfb:A	1o7j:A	1hzt:A	1osp:O
1jg1:A	1obd:A	1io0:A	1qdd:A
1jhd:A:1–173	1pin:A:6–39	1iv3:A	1qhd:A:1–148
1jz7:A:220–333	1qjb:A	1iw0:A	1vhh
1k20:A	1slu:A	1j96:A	1vps:A
1k5n:A:182–276	1uaq:A	1j9j:A	1wer
1k92:A:189–444	1uca:A	1jf8:A	2bop:A
1kg2:A	1uxy:201–342	1jl0:A	2nac:A:1–147
1kgs:A:124–225	2sic:I	1k3w:A:1–124	3lzt
1ko7:A:1–129	1a9x:A:403–555	1k5c:A	3seb:122–238
1kr4:A	1axn	1kid	

It is important to point out here that the randomization of sequences in native structures is done only to provide sets of 50 random decoys each for the first-level test, while the reversal of sequences is done on native structures in the training dataset to provide the neural networks with a number of native-like negative training examples equal in size and number to the native structures, which are the positive training examples. It is previously mentioned in the case of generated structures of reversed sequences that structural features such as pairwise distances are retained, and sequence-related features such as the distance distributions of pairwise residues have been altered. The same applies to generated structures with randomized sequences, except that the distributions of pairwise distances of such structures would be more different to those of native structures. This is because in the case of generated structures with reversed sequences, information regarding the proximity between certain residues along the sequence is retained. For example, an Alanine pair of 6 residues apart which can be found in a helix in the native structure would still be 6 residues apart in the reversed structure but they may be part of a beta strand or loop. This type of information would be lost in the randomized structures.

The purpose of preliminary testing is to test the viability of the idea of using neural networks in the current context for decoy discrimination, which includes the paradigm of using structures with reversed sequences as negative training examples, and the separation of native from random decoy structures as a quick first-level test. Using the NN-dist method and the S combination of network results, 91 out of 95 native structures are correctly ranked with the highest network scores. This means that for the 95 native structures in the preliminary test dataset and the 50 corresponding random decoys for each native structure, the S combination of the NN-dist method is able to assign the highest network score to the native structure for 91 cases. The average Z score of these 95 proteins is 3.851. The remaining 4 cases have their native structures ranked 2^nd^, 3^rd^, 8^th ^and 23^rd ^out of 51 structures (native+50 random structures). Apart from the worst performing native structure, 1vps:A, the native structures of the other 3 cases have comparatively high ranks, even though they do not have the highest rank. The protein domain 1vps:A has 4 other similar domains, and forms part of a beta sandwich architecture, and the poor Z score of its native structure could be due to the fact that 22 generated structures with randomized sequences have, due to pure chance, distributions of pairwise distances similar to that of native distributions.

### Results of methods with no evolutionary information

#### Results of different combinations of scores

**Figure 1:**
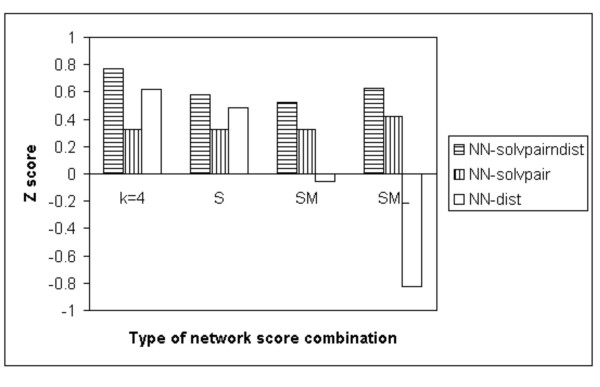
Z scores produced by the NN-solvpairndist, NN-solvpair and NN-dist methods on all the proteins in the Baker decoy dataset across the different *k *= 4, S, SM and SML combinations.

Figure 1 shows the different Z scores obtained by the NN-dist, NN-solvpair and NN-solvpairndist methods on the Baker dataset [16]. The single *k *= 4 mean score is compared with the S, SM and SML combinations of network scores.

It can be seen from Figure 1 that there is little difference between the different combinations of network scores for the NN-solvpair method. For the NN-solvpairndist method, the *k *= 4 single mean score is better than the other combinations. For the NN-dist method, there is more variability between the different combinations, with the SM and SML combinations yielding negative Z scores. Figure 1 also shows that the NN-solvpairndist method performs better than the other two methods across all different combinations of scores.

Due to the fact that the NN-dist method does well for the S combination, and the rest of the methods perform rather consistently across the different combinations, the S combination is used for benchmarking for all decoy datasets in the remaining results presented in this work. Although the *k *= 4 single mean score yields the highest Z score for the NN-solvpairndist and NN-dist methods on the Baker dataset [16], it is poorer than the S combination in most of the other decoy datasets (data not shown).

#### Results on Baker dataset

**Figure 2:**
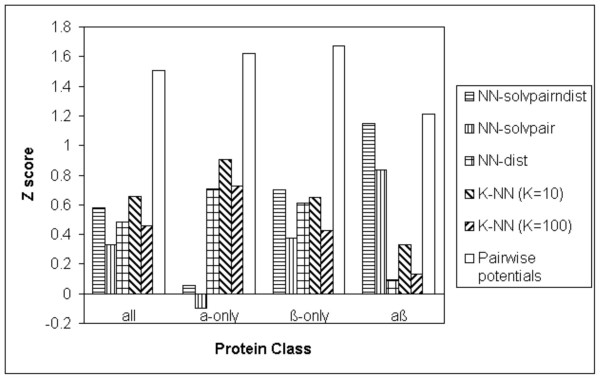
Z scores produced by the S combination of the NN-solvpairndist, NN-solvpair, NN-dist methods, the K Nearest Neighbours methods (K = 10, K = 100) and the pairwise potentials method on the different secondary structural classes of the Baker decoy dataset.

Because the Baker dataset [16] has 22 proteins, it is possible to see how the various methods, including the pairwise potentials [17] and the K Nearest Neighbours method, perform on different secondary structural classes of proteins. Figure 2 shows the Z scores produced by the S combination of the various neural network and K Nearest Neighbours methods on the different secondary structural classes of proteins in the Baker dataset [16].

It can be seen from Figure 2 that for the S combination, the NN-solvpairndist and NN-solvpair methods do not perform well for α-only proteins in the Baker decoy dataset [16], compared to the rest of the methods. The reverse is true for αβ proteins where the NN-solvpairndist and NN-solvpair methods have higher Z scores than the NN-dist method and the K Nearest Neighbours methods. In all cases, the pairwise potentials method [17] has the highest Z score and it is interesting to note that the NN-solvpairndist method has a Z score which is only marginally lower than that of the pairwise potentials method [17] for αβ proteins.

The NN-dist method and the K Nearest Neighbours methods (K = 10, 100) perform well for α-only and β-only proteins, but has low Z scores for αβ proteins. The K = 10 method performs slightly better than the K = 100 method for all classes.

On average, across all proteins, the pairwise potentials method [17] has the highest Z score. The NN-solvpairndist method performs slightly better than the NN-dist method, while the K Nearest Neighbours method (K = 10) has an overall Z score which is slightly higher than the NN-solvpairndist method.

#### Results on all datasets

**Figure 3:**
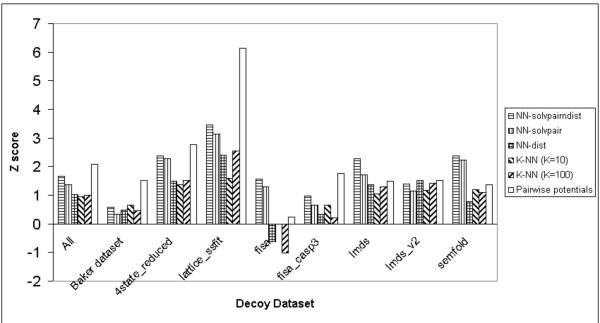
Z scores produced by the S combination of the NN-solvpairndist, NN-solvpair, NN-dist methods, the K Nearest Neighbours methods (K = 10, K = 100) and the pairwise potentials method on the different individual decoy datasets, including the combination of all the individual datasets.

Figure 3 shows the Z scores obtained on all decoy datasets with the NN methods (with no evolutionary information), the pairwise potentials method [17] and the K Nearest Neighbours methods. For the combination of all datasets, the pairwise potentials method [17] has the highest Z score, while the NN-solvpairndist method has the second highest Z score.

In general, although the pairwise potentials method [17] has the highest overall Z score, it does not have the highest Z score for each individual dataset. For the fisa [4], lmds [22] and semfold [23] datasets, the NN-solvpairndist method has the highest Z score instead. The NN-solvpairndist method also has the second highest Z score after the pairwise potentials method [17] in the 4state_reduced [24], lattice_ssfit [25] and fisa_casp3 [4] datasets. This suggests that the NN-solvpairndist method shows some promise in matching the performance of the pairwise potentials method [17], if it can be further augmented with additional information.

In all but one case (lmds_v2 [22]), the NN-solvpairndist method has a higher Z score than the NN-dist method. For all but 2 decoy datasets (Baker [16] and lmds_v2 [22]), the 2 K-NN methods have lower Z scores than the NN-solvpairndist method, although they are comparable to the NN-dist method in terms of Z score. The NN-solvpair method also performs better than the NN-dist method in all but two cases, namely the Baker decoy dataset [16] and lmds_v2 [22]. The NN-solvpairndist method always has higher Z scores than the NN-solvpair method, which suggests that the additional distance information of the NN-solvpairndist method contributes to the discrimination of native structures.

**Figure 4:**
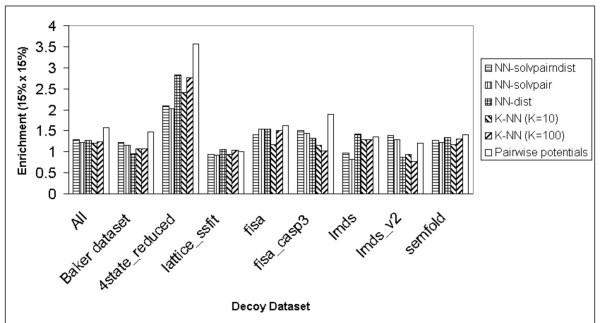
Enrichment scores (15% × 15%) produced by the S combination of the NN-solvpairndist, NN-solvpair, NN-dist methods, the K Nearest Neighbours methods (K = 10, K = 100) and the pairwise potentials method on the different individual decoy datasets, including the combination of all the individual datasets.

Figure 4 shows the enrichment scores [16] of the S combination across all decoy datasets for the different methods. For the combined datasets, the pairwise potentials method [17] has the highest enrichment score [16], while the NN-solvpairndist method is comparable to the rest of the other methods. For most of the decoy datasets, there is no clear outstanding method which produces a distinctly high enrichment score [16], apart from the pairwise potentials method [17] in the Baker [16], 4state_reduced [24] and fisa_casp3 [4] datasets.

It also seems that there is no significant improvement of the enrichment score [16] for the NN-solvpairndist method over the NN-dist method.

#### Results of methods using evolutionary information

In this section, the results of the sequence profile methods and homologue threading methods are presented. Because the semfold [23] dataset has about 11000 decoys per protein, and there is a lack of computational resources during the application of the homologue threading methods for the threading of such a large number of decoy structures, the semfold [23] dataset is left out in this section. The K Nearest Neighbours methods are also left out to increase the clarity of the graphs.

**Figure 5:**
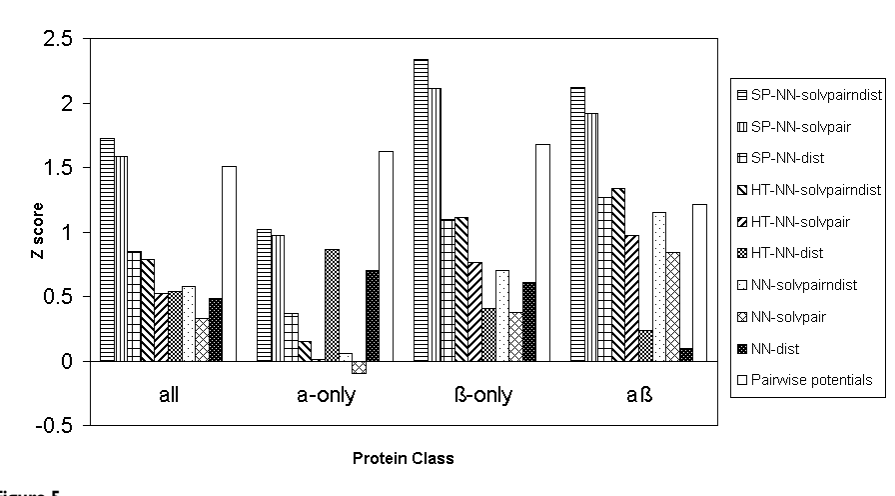
Z scores produced by the S combination of the sequence profile (SP) methods, the homologue threading (HT) methods, the basic NN-solvpairndist, NN-solvpair, NN-dist methods, and the pairwise potentials method on the different secondary structural classes of the Baker decoy dataset.

Figure 5 shows the Z scores produced by the S combination of the sequence profile (SP) methods, the homologue threading (HT) methods, the basic NN-solvpairndist, NN-solvpair and NN-dist methods, as well as the pairwise potentials method [17], on the different secondary structural classes of proteins from the Baker dataset [16]. It can be seen that the SP-NN-solvpairndist and SP-NN-solvpair methods experience a significant increase in Z score over their non-evolutionary counterparts. For the α-only proteins, the pairwise potentials method [17] is still the best, but overall, the SP-NN-solvpairndist method has the highest Z score.

It can also be observed that the homologue threading methods has higher Z scores than the non evolutionary counterparts across all classes, except β-only, of proteins. This suggests that there is a small but noticeable reduction in noise in the discrimination of native structures when the averaging of network output scores of sequence homologues is used.

**Figure 6:**
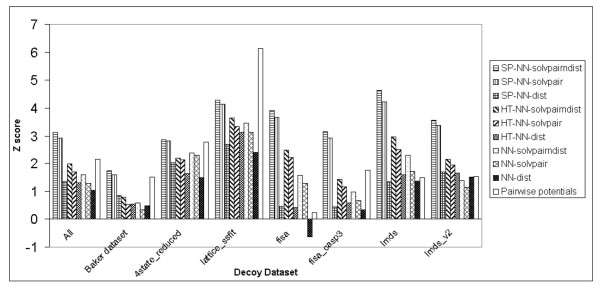
Z scores produced by the S combination of the sequence profile (SP) methods, the homologue threading (HT) methods, the basic NN-solvpairndist, NN-solvpair, NN-dist methods, and the pairwise potentials method on the different individual decoy datasets, including the combination of all the individual datasets.

Figure 6 shows the key result of this paper, where the SP-NN-solvpairndist and SP-NN-solvpair methods clearly outperform all other methods in the discrimination of native structures for all decoy datasets apart from the lattice_ssfit [25] dataset. In Figure 3, the pairwise potentials method [17] is the best for the fisa_casp3 [4] dataset, but the SP-NN-solvpairndist and SP-NN-solvpair methods clearly outperform it, as shown in Figure 6. Among these two methods, the SP-NN-solvpairndist method has a slightly higher Z score than the SP-NN-solvpair method in all datasets.

**Figure 7:**
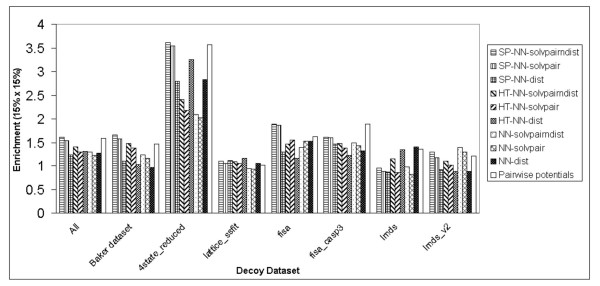
Enrichment scores (15% × 15%) produced by the S combination of the sequence profile (SP) methods, the homologue threading (HT) methods, the basic NN-solvpairndist, NN-solvpair, NN-dist methods, and the pairwise potentials method on the different individual decoy datasets, including the combination of all the individual datasets.

In Figure 7, for the SP-NN-solvpairndist method, the performance on the enrichment score [16] for the S combination is less pronounced than that of the Z score. The SP-NN-solvpairndist method ranks best in the Baker decoy dataset [16], 4state_reduced [24], and fisa [4] datasets.

In Figure 6, for the HT-NN-dist, HT-NN-solvpair and HT-NN-solvpairndist methods, apart from the 4state_reduced [24] dataset, it can be seen that the averaging of sequence homologues do yield a slight increase of Z score for each HT-NN method over its corresponding basic NN counterpart method. This suggests that a modest increase in the performance of the discrimination of native structures can be achieved using averaging the scores of sequence homologues that are threaded to each structure in the decoy dataset. Table 6 shows the number of homologous sequences produced by PSI-BLAST [1] for the native proteins in the various decoy datasets.

**Table 6:** Number of homologues for the proteins in the decoy datasets for the homologue threading methods

**Decoy Dataset**	**Number of proteins**			
	
	**Total**	**with no homologues**	**with > = 10 homologues**	**With <10 homologues**
Baker	22	5	14	3
4state_reduced	6	0	6	0
lattice_ssfit	8	1	7	0
fisa	4	0	3	1
fisa_casp3	4	1	3	0
lmds	10	1	7	2
lmds_v2	10	0	7	3

For the enrichment score [16], the HT-NN methods show little improvement over the basic counterpart methods. All the 3 homologue threading methods show improvements over the basic NN methods in only 3 datasets, namely the Baker dataset [16], 4state_reduced [24] and the lattice_ssfit [25] datasets.

One conclusion that can be drawn is that the SP-NN-solvpairndist method, which uses profile information in conjunction with pairwise distance and relative solvent accessibility information of residue pairs, has the best performance in terms of the discrimination of native structures for all decoy datasets (Z score) among the various neural network methods and the pairwise potentials method [17]. In terms of selecting the low RMSD decoys (enrichment score [16]), it slightly outperforms the rest of the methods for a number of decoy datasets.

#### Results of statistical tests

This section discusses the results of two statistical tests, the first of which evaluates the difference between two decoy discrimination methods in the ability to select the top ranked model of highest quality. The assessment of the quality of the top ranked model itself is done using different structural similarity measures, namely TM-score [19], GDT-TS [20] and MaxSub [21]. The one-tailed Wilcoxon sign-rank test is used here to test, at a 5% significance level, the null hypothesis of zero median in the distribution of differences in structural similarity scores of the respective top ranked models produced by the two methods.

The second statistical test compares the differences between the ability of the two decoy discrimination methods to assign high scores to better quality models and low scores to poorer quality models. The Spearman rank correlation coefficient is used here to measure the correlation between the output scores of each method and the structural similarity (e.g. TM-score[19]) score of the decoy structures. The one-tailed Wilcoxon sign-rank test is then used in the assessment of the difference, if any, in the distributions of the Spearman rank correlation coefficients produced by both methods.

Table 7 shows the *p*-values of the one-tailed Wilcoxon sign-rank tests in top model selection between the SP-NN-dist, SP-NN-solvpair and SP-NN-solvpairndist methods and their corresponding basic NN methods, namely NN-dist, NN-solvpair and NN-solvpairndist respectively. All the individual decoy datasets are tested, along with the combined dataset, which is also subdivided into α-only, β-only and αβ classes of proteins. A *p-*value of ≤ 0.05 means that the null hypothesis of zero median in the distribution of differences between the structural similarity measures of the respective top ranked models can be rejected at 5% significance level.

**Table 7:** Top model selection: P-values of one-tailed Wilcoxon sign-rank test between the SP-NN-dist, SP-NN-solvpair, SP-NN-solvpairndist methods and the corresponding basic NN methods

**Decoy Dataset**	**SP-NN-dist**			**SP-NN-solvpair**			**SP-NN-solvpairndist**		
	
	**NN-dist**			**NN-solvpair**			**NN-solvpairndist**		
	
	**TM-score**	**GDT-TS**	**MaxSub**	**TM-score**	**GDT-TS**	**MaxSub**	**TM-score**	**GDT-TS**	**MaxSub**
4state_reduced	0.1562	0.1562	0.1562	0.0938	0.0938	0.0938	0.1562	0.1562	0.1562
Baker	0.8474	0.8743	0.6553	**0.0430**	**0.0430**	0.0727	**0.0366**	**0.0337**	0.0502
fisa_casp3	0.0625	0.0625	0.0625	0.5625	0.5625	0.3125	0.5	0.5	0.5
fisa	0.5	0.625	0.625	0.5	0.5	0.5	0.625	0.5	0.625
lattice_ssfit	0.3711	0.7266	0.5	0.1875	0.5312	0.0781	0.1914	0.7266	0.1562
lmds	0.9863	0.9941	0.9512	0.7695	0.3711	0.6289	0.7871	0.2852	0.8496
lmds_v2	0.2158	0.2783	0.5449	0.2891	0.3438	0.4219	0.918	0.8984	0.8203
semfold	0.8906	0.9219	0.9219	0.3125	0.3125	0.1875	0.9375	0.5	0.8438
All	0.7256	0.8031	0.6942	0.0651	**0.0258**	**0.0370**	0.2048	0.0926	0.1966
αβ	0.2877	0.574	0.8428	0.1906	0.8863	0.2507	0.9781	0.9886	0.956
α-only	0.8408	0.8895	0.7858	0.0614	**0.0185**	**0.0432**	**0.0438**	**0.0115**	**0.0490**
β-only	0.3438	0.6562	0.0781	0.1562	0.1562	0.6289	0.1562	0.1562	0.6289

In Table 7, the comparison of the SP-NN-solvpairndist method with the NN-solvpairndist method is shown to have p-values ≤ 0.05 for the α-only set of proteins for all structural similarity measures, and hence the null hypotheses can be rejected in these cases. This suggests that evolutionary information, in the context of the neural network that uses relative solvent accessibilities and pairwise distance, can yield better quality top ranked models in α-only proteins.

Table 8 repeats the same tests for the ranking of Spearman correlation coefficients between the SP-NN-dist, SP-NN-solvpair and SP-NN-solvpairndist methods and their corresponding basic NN methods, namely NN-dist, NN-solvpair and NN-solvpairndist respectively. A *p-*value of ≤ 0.05 means that the null hypothesis of zero median in the distribution of differences between the Spearman correlation coefficients of the structural similarity measures and the output scores of the two methods can be rejected at 5% significance level.

**Table 8:** Spearman correlation coefficient: P-values of one-tailed Wilcoxon sign-rank test between the SP-NN-dist, SP-NN-solvpair, SP-NN-solvpairndist methods and the corresponding basic NN methods

**Decoy Dataset**	**SP-NN-dist**			**SP-NN-solvpair**			**SP-NN-solvpairndist**		
	
	**NN-dist**			**NN-solvpair**			**NN-solvpairndist**		
	
	**TM-score**	**GDT-TS**	**MaxSub**	**TM-score**	**GDT-TS**	**MaxSub**	**TM-score**	**GDT-TS**	**MaxSub**
4state_reduced	0.5781	0.5781	0.5781	**0.0156**	**0.0156**	**0.0156**	**0.0156**	**0.0156**	**0.0156**
Baker	0.2961	0.2529	0.2961	**0.0003**	**0.0001**	**0.0008**	**0.001**	**0.0004**	**0.0017**
fisa_casp3	0.3125	0.1875	0.4375	0.125	0.125	0.125	0.0625	0.0625	0.0625
fisa	0.0625	0.0625	0.0625	0.125	0.125	0.125	0.125	0.125	0.125
lattice_ssfit	0.2305	0.2305	0.3711	0.0977	0.2305	0.0547	0.125	0.2305	0.0977
lmds	0.5771	0.8389	0.7217	0.0967	0.0527	0.1377	0.5	0.3477	0.4229
lmds_v2	0.7217	0.6875	0.7217	0.4229	0.7217	0.4609	0.3848	0.4609	0.4609
semfold	0.0781	0.1094	0.0781	0.0781	0.0781	**0.0469**	0.2812	0.1562	0.1562
All	0.109	0.1157	0.1508	**1.90e-07**	**2.50e-07**	**3.90e-07**	**6.40e-06**	**4.30e-06**	**6.60e-06**
αβ	0.0727	0.1567	0.0584	**0.0062**	**0.0032**	**0.0029**	**0.0261**	**0.02**	**0.0337**
α-only	0.5853	0.5234	0.8412	**0.0001**	**0.0001**	**0.001**	**0.0018**	**0.0008**	**0.002**
β-only	**0.0391**	**0.0391**	**0.0391**	**0.0391**	**0.0391**	**0.0391**	**0.0273**	**0.0195**	**0.0273**

In Table 8, the comparison of the SP-NN-solvpairndist method with the NN-solvpairndist method is shown to have *p-*values ≤ 0.05 for the combined dataset of proteins, as well as the α-only, β-only, αβ classes of proteins, for all structural similarity measures. The same can be said of the comparison between the SP-NN-solvpair and NN-solvpair methods too. Hence the null hypotheses can be rejected in these cases. This suggests that additional evolutionary information, in the context of the SP-NN-solvpair and SP-NN-solvpairndist methods, can produce better Spearman correlation coefficients between the quality of the models and the output scores.

Table 9 shows the comparison of the SP-NN-solvpairndist method with the in-house pairwise potentials method[17] on both the ability to select good quality top-ranked models and to produce good Spearman correlation coefficients between the output scores and TM-scores [19]. It can be seen for the Baker dataset[16] that the hypothesis of zero median in the distribution of differences between the Spearman correlation coefficients of TM-score[19] and the output scores of the two methods can be rejected at 5% significance level. However for top model selection in the Baker dataset [16], the null hypothesis can only be rejected at 10% significance level. This means that at 10% significance level, we can reject the hypothesis that the SP-NN-solvpairndist method is not better than the in-house pairwise potentials method[17] in terms of producing better quality, as measured by TM-score [19], top-ranked models.

**Table 9:** P-values of one-tailed Wilcoxon sign-rank test between SP-NN-solvpairndist method and the in-house pairwise potentials method, with TM-score as the structural similarity measure, for top model selection and Spearman correlation.

**Decoy Dataset**	**Top Model Selection**	**Spearman correlation**
4state_reduced	0.5781	0.5000
Baker	**0.0838**	**0.0412**
fisa_casp3	0.5625	0.4375
fisa	0.9375	0.4375
lattice_ssfit	0.6797	0.1250
lmds	0.8838	0.9346
lmds_v2	0.8125	0.6523
semfold	0.9844	0.9219
All	0.8612	0.4131
αβ	0.9164	0.6994
α-only	0.8364	0.4070
β-only	0.3203	0.2734

#### Comparison with Dong's profile-based statistical potentials

One existing method that uses evolutionary information for discriminating native structures from decoy datasets is developed by Dong and co-workers [15] in the form of profile-based statistical potentials. In their work, they extend the traditional paradigm of residue identity in pairwise potentials [17] to that of frequency profiles, which represent the probabilities of amino acids occurring in specific positions of protein sequences. These frequency profiles are converted to binary profiles via probability thresholds. A variety of profile level statistical potentials, including distance-dependent, contact, dihedral angle and accessible surface potentials, are created and tested on various decoy datasets. In this paper, we compare the Z scores of our most promising method, SP-NN-solvpairndist, to that of the most successful method, the distance profile method, of Dong and co-workers[15]. Table 10 shows the Z scores applied to the various decoy datasets. The number of protein decoy datasets used in our method and Dong's method differs for the Baker decoy dataset[16], the 4state_reduced [24] and fisa_casp3[4] datasets. For example, we use only the 22 non-obsolete X-ray structures in the Baker dataset[16], while Dong and co-workers used the whole set of 41 structures. Similarly, for the 4state_reduced[24] dataset, we use 6 non-obsolete protein decoy datasets instead of 7 and Dong used 3 instead of 4 protein decoy datasets in fisa_casp3[4].

**Table 10:** Comparison of Z scores of Dong's profile statistical potentials and the SP-NN-solvpairndist method on decoy datasets.

Dataset\Method	In-house pairwise potentials	SP-NN-solvpairndist	Dong's Distance_profile	Dong's Distance
Baker	1.51 (22)	1.72 (22)	2.74 (41)	2.58 (41)
4state_reduced	2.78 (6)	2.86 (6)	3.53 (7)	2.48 (7)
lattice_ssfit	6.13 (8)	4.29 (8)	5.72 (8)	4.97 (8)
lmds	1.48 (10)	4.64 (10)	2.45 (10)	1.78 (10)
fisa	0.24 (4)	3.92 (4)	3.32 (4)	3.06 (4)
fisa_casp3	1.75 (4)	3.16 (4)	2.94 (3)	1.93 (3)

For the Baker decoy dataset [16], Dong's statistical profile potentials has a higher Z score than the SP-NN-solvpairndist method, but the Z score is derived over additional low-resolution NMR native structures in the Baker dataset[16] for the former method. For the 4state_reduced[24] and lattice_ssfit[25] datasets, Dong's distance profile method has higher Z scores than the SP-NN-solvpairndist method, although the 4state_reduced[24] dataset used by Dong and co-workers include one additional obsolete structure. However, our SP-NN-solvpairndist method has higher Z scores than Dong's distance profile method for identical number of proteins in the lmds[22] and fisa[4] datasets, as well as for the fisa_casp3[4] dataset, where the number of protein decoy datasets differ.

## Discussion

In this paper, we introduce the idea of replacing sets of mean force potentials by a small number of neural networks to perform discrimination of native structures from decoys. Different combinations of input features were tried, with the most useful features being pairwise distance between residue pairs, solvent accessibility values of the residues, and/or evolutionary information encoded in the form of profiles.

Positive training examples are extracted from a set of native protein structures, and negative training examples are simulated using the sequence reversal method on the same set of protein structures. Testing is done on several publicly available decoy datasets, and results are compared to that obtained using the in-house traditional pairwise potentials method [17].

Results show that as far as the discrimination of the native structure is concerned, neural networks without evolutionary information do not perform as well as statistical potentials across the various datasets (Figure 3). The best neural network has a Z score of 1.66 compared to the pairwise potentials method[17]'s Z score of 2.07. However, the neural network with the input combination of pairwise distance and solvent accessibilities does as well for the αβ class of proteins in the Baker dataset[16] (Figure 2), with a Z score of 1.15 compared to the Z score of 1.21 produced by the pairwise potentials method [17]. For the enrichment score [16], the best neural network without evolutionary information has a score of 1.29, compared to the pairwise potentials method's enrichment score [16] of 1.57 (Figure 4).

However, addition of evolutionary information helps improve the discrimination of the native structure considerably, as measured by the Z score, compared to statistical pairwise potentials[17] (Figure 6). The best neural network, which comprises a combination of all input features, has a Z score of 3.12 compared to the pairwise potentials method's Z score of 2.14 (This Z score is different from the previous stated Z score of 2.07 due to the omission of the semfold[23] decoy dataset, as explained earlier). For the different classes of proteins in the Baker dataset [16], the same neural network outperforms the pairwise potentials method[17] for the β-only and αβ classes, but not the α-only class of proteins. The enrichment score [16] of the best network utilizing evolutionary information is 1.62, a marginal improvement over the statistical pairwise potentials' score of 1.59 (Figure 7).

It is mentioned in the work of Park and co-workers[26] that different decoy discrimination functions work well in some datasets, but not in others due to the differences in the construction of decoy datasets. From Figure 6, the best neural network, SP-NN-solvpairndist, appears to have similar levels of performance for the various decoy datasets, apart from the Baker dataset [16] which is highest in quality. Figure 5 however shows that the SP-NN-solvpairndist neural network performs better with β-only and αβ classes of proteins, as compared to the α-only class of proteins. This suggests that this neural network can be used in conjunction with the in-house pairwise potentials method [17], which performs well for α-only proteins, for decoy discrimination, depending on the predicted secondary structural content of the target sequence.

The use of neural networks in decoy discrimination yield mixed results, with much better results achieved from those neural networks that use evolutionary information. This suggests that the sequence reversal method of generating negative training examples is, on the whole, feasible and works better in those neural networks that utilise evolutionary information. This is not surprising because evolutionary information in the form of PSI-BLAST [1] profiles encode information regarding the affinity of all possible residue types to occupy two particular positions in the sequence. For the native structures that represent positive training examples, such information represented by the multi-dimensional distributions of features such as the pairwise distance can be effectively encoded by the neural networks. Similarly, for the generated structures with reversed sequences that represent the negative training examples, the distributions representing the affinities of residue types to any two "false" positions, brought about by the reversal of sequence, have a stronger negative signal compared to that of the non-evolutionary information, and this can be effectively encoded by the neural networks in the training phase.

## Conclusion

In this paper, we have demonstrated the viability of using machine learning, more specifically neural networks, to perform decoy discrimination.

The method of simulating the decoy structures using the sequence reversal method appears to provide an adequate representation of negative training examples in neural network learning. A combination of input features of the identities of the residue pairs, pairwise distance, and relative solvent accessibility information proves promising in the discrimination of native structures. We also show that evolutionary information can be used to further improve the discrimination process.

The best neural network method (SP-NN-solvpairndist) has input features comprising of the position-specific sequence profile information of residue pairs, together with the relative solvent accessibility of the residues and the pairwise distance between these residues. The SP-NN-solvpairndist method is the best among all the methods tested in discriminating native structures from the corresponding set of decoy structures, as demonstrated by the highest Z scores it has in all the decoy datasets in Figure 6. As for the enrichment [16] in Figure 7, the SP-NN-solvpairndist method is the best in approximately half of the decoy datasets tested.

Statistical tests have shown at a 5% significance level that, for α-only proteins, the SP-NN-solvpairndist method can select a top model of better quality, using structural similarity measures of TM-score[19], GDT-TS[20] or MaxSub [21], than the corresponding NN-solvpairndist method. This suggests that evolutionary information can help to increase the quality of top model selection.

At a 5% significance level, for the combined dataset and the various secondary structural classes of proteins, there is an increase in the Spearman correlation coefficients of the structural similarity measures of decoy models with the output scores of the SP-NN-solvpairndist and SP-NN-solvpair methods, when compared to the Spearman correlation coefficients of the structural similarity measures with the output scores of the corresponding basic NN-solvpairndist and NN-solvpair methods respectively. This again suggests that evolutionary information helps to better correlate the quality of the decoy models with the output scores of the proposed decoy discrimination method.

In conclusion, the idea of applying machine learning for the decoy discrimination problem, in context of using neural networks and the proposed way of representing the required training examples, is indeed feasible, as demonstrated in this paper. Furthermore, decoy discrimination, in particular the identification of the native structure, can be greatly improved by using evolutionary information in the form of including PSI-BLAST [1] profiles in the training of the neural networks.

A major advantage of this approach to evaluating protein models is that any number of relevant features can be directly taken account of in the formulation. Here we have looked primarily at evolutionary information, but information from experimental sources (e.g. from circular dichroism or NMR chemical shifts) could also be incorporated.

Further improvements to decoy discrimination can be expected through the use of better simulated decoy structures. Rather than using crude reversed sequence threading models, a large ensemble of refined *de novo *models could be used, or perhaps decoy structures specifically tuned to particular sizes of proteins or folding types, for example.

A future variation of this idea will be to extend this machine learning paradigm to high resolution modelling which involves the discrimination of close-to-native decoys from native structures. Although the idea of using reversed sequences to provide negative training examples works reasonably well for presently available low-resolution decoy datasets, it cannot be applied to the discrimination of native structures from close-to-native decoys. In this case, suitable models for the negative training examples could be taken from sets of comparative models built from templates of closely related structures[27].

## Methods

### Dataset of protein domains

A large and diverse dataset of protein domains is required for neural network training and validation, as well as testing. The validation subset is necessary to prevent over-fitting during neural network training, and the test dataset is used for preliminary testing. In this work, this dataset is obtained from the SCOP database [18] and subsequently partitioned into 3 parts. The initial unpartitioned dataset is referred to as the 'initial dataset' in the remainder of this section.

The proteins in the initial dataset are chosen to be structurally non-homologous to one another. This means that no two pairs of protein domains in the initial dataset is structurally similar, in the context of SCOP's classification method [18]. This is done to facilitate the partitioning of the validation and test datasets from the initial dataset. If all pairs of protein domains are non-homologous, there is no cause for worry of homologous proteins existing between the training and validation/test datasets when randomly assigning proteins to the validation/test datasets during the partitioning process.

To create such a initial dataset, the SCOP[18] domain database (v1.65, December 2003) is used. One domain from each superfamily of SCOP classes *a *to *d *is selected. There are altogether 1095 superfamilies in these 4 SCOP classes. For each superfamily, the first domain is chosen that is an X-ray structure, has a resolution of 2Å or better, and is not part of a protein whose domain has already been selected. Because many sparsely-populated superfamilies have no domains whose criteria are met, many superfamilies do not contribute to the initial dataset and the number of this initial set of domains is 740.

However, 28 proteins in this initial set of domains share the same superfamily with at least one of the proteins in the decoy datasets, namely the Tsai decoy dataset (referred to as Baker dataset [16] in the text) and the Decoys 'R' Us suite of decoys[28] (to be elaborated in following sections). Therefore these 28 proteins are excluded from the initial dataset. Furthermore, 265 proteins from this initial set (after the 28 proteins have been excluded) have less than 10 sequences in the multiple sequence alignments after PSI-BLAST [1] is run, and these are excluded from the training dataset. Strictly speaking, there is no need to exclude these 265 proteins from the training dataset when training neural networks with single sequences (without evolutionary information). However to facilitate the comparison of results obtained from training with single sequence information to results obtained from the inclusion of evolutionary information during neural network training, it would be more precise if the set of training data is being kept constant. The final set of protein domains, after both exclusion steps, has 475 proteins.

This final dataset of 475 protein domains is then divided into 3 parts, namely 60% for the training dataset, 20% for validation and 20% for preliminary testing. Tables 11, 12 and 5 show the training set of 285 protein domains, the validation set of 95 protein domains and preliminary test data of 95 protein domains respectively. All 3 datasets have mixtures of secondary structural classes, as shown in Table 1. From Table 1, it can be seen that αβ proteins are about twice the number of α-only and β-only proteins because the αβ class in Table 7 consists of proteins from SCOP classes *c *and *d*, while the α-only and β-only proteins come from SCOP classes *a *and *b *respectively.

**Table 11:** Training dataset of 285 proteins

**Training Dataset : Protein {:Chain}{:Domain Boundaries}**				
1a6q:297–368	1lok:A	1etx:A	1nj4:A:263–355	1egw:A
1ako	1lqp:A	1ew4:A	1nls	1erz:A
1ayl:228–540	1ltz:A	1eyq:A	1nzi:A:1–117	1ftr:A:1–148
1ayo:A	1m1n:A	1eyv:A	1o08:A	1gpr
1bx4:A	1mf7:A	1f60:A:241–334	1o1x:A	1gxu:A
1byq:A	1mgp:A	1g61:A	1oi7:A:122–288	1h16:A
1c5k:A:35–162	1mn8:A	1gs9:A	1qnf:205–475	1h4x:A
1c97:A:2–528	1mv8:A:203–300	1gso:A:-2–103	1qre:A	1hqs:A
1cip:A:61–181	1nf9:A	1hbn:A:2–269	1qtn:A	1hs6:A:1–208
1cuk:156–203	1nvm:A:291–341	1hx0:A:404–496	1who	1hz4:A
1d8c:A	1o98:A:77–310	1i1w:A	2pth	1jid:A
1ejx:A	1oo0:A	1iom:A	2uag:A:298–437	1k4g:A
1evl:A:533–642	1orv:A:39–508	1j3a:A	3grs:364–478	1kj9:A:113–318
1f46:A	1p3d:A:107–321	1jat:A	4eug:A	1kq1:A
1fye:A	1qhp:A:577–686	1jhf:A:73–198	16pk	1kqf:A:34–850
1g8l:A:327–409	1qna:A:17–115	1jkx:A	1bkr:A	1dfu:P
1gdn:A	1rl6:A:7–81	1k3x:A:125–213	1c8z:A	1mwx:A:139–327
1gpj:A:303–404	1tfe	1k3y:A:81–222	1cs0:B:2–152	1p5u:A:148–234
1h4a:X:1–85	4ubp:B	1k7k:A	1dg6:A	1qop:A
1heu:A:164–339	1af7:11–91	1kbl:A:377–509	1di6:A	1whi
1hp1:A:363–550	1aie	1kmt:A	1dmh:A	1xxa:A
1hqk:A	1bd8	1lam:1–159	1dqe:A	2ilk
1ir1:S	1cqm:A	1ld8:A	1duv:G:1–150	2sns
1kb0:A:1–573	1dl2:A	1m15:A:2–95	1dw9:A:87–156	2spc:A
1kqp:A	1dto:A	1me4:A	1dy5:A	3nul
1l8a:A:701–886	1e39:A:360–505	1me8:A:2–101	1e58:A	8ruc:A:9–147
1l8b:A	1e4c:P	1mg4:A	1eaz:A	1a77:209–316
1ldg:164–329	1ekr:A	1moo:A	1ez3:A	1a8o
1lkk:A	1elk:A	1muw:A	1f8n:A:6–149	1c1d:A:1–148
1lqt:A:2–108	1exm:A:313–405	1mvl:A	1fdr:101–248	1crz:A:141–409
1m1g:A:132–190	1fwx:A:8–451	1n55:A	1fsg:A	1czp:A
1m26:A	1gmi:A	1n60:B:7–146	1fx2:A	1dci:A
1mj4:A	1gs5:A	1n61:C:1–177	1g8m:A:4–200	1mfm:A
1n1b:A:271–598	1gtk:A:220–313	1n8k:A:1–163	1gkm:A	1g7s:A:329–459
1n3l:A	1hf8:A	1nm8:A:9–385	1gmx:A	1gkp:A:2–54
1n62:A:82–163	1hlr:A:311–907	1o26:A	1got:G	1gxr:A
1ns5:A	1hq1:A	1ofd:A:1240–1507	1gqz:A:1–130	1hxk:A:31–411
1nxj:A	1ijy:A	1oht:A	1gvo:A	1i9c:A
1oe1:A:1–159	1ix9:A:1–90	1or7:A:-1–111	1h05:A	1iu7:A:212–628
1p5v:A:7–147	1iz5:A:2–120	1ox0:A:-5–251	1h3n:A:226–417	1j8b:A
1qhv:A	1jos:A	1qcz:A	1hb6:A	1jp3:A
1qqf:A	1k0r:A:184–262	1qh4:A:103–381	1i2t:A	1jsd:A
1qsa:A:1–450	1khb:A:10–259	1qnx:A	1i4j:A	1jw9:B
1rss	1khd:A:12–80	1d0c:A	1ikt:A	1k8y:B
1tig	1kp8:A:2–136	1dd3:A:1–57	1ixb:A:91–205	1kek:A:416–668
1yge:150–839	1krh:A:106–205	1dj0:A:7–114	1ixh	1kpf
2tps:A	1kwm:A:1A-95A	1dow:A	1j6z:A:4–146	1ku1:A
7odc:A:44–283	1kyp:A	1dqi:A	1jke:A	1mc2:A
1awq:A	1l6p:A	1e6i:A	1k4i:A	1lb3:A
1bd0:A:2–11	1mix:A:195–308	1ei5:A:336–417	1k6d:A	1m9n:A:201–593
1bxy:A	1mla:198–307	1f86:A	1knl:A	1mgt:A:89–169
1byi	1n5u:A:2–196	1f9y:A	1l2h:A	1nkp:A
1chd	1np7:A:1–204	1fhu:A:1–99	1l5o:A	1npk
1cxq:A	1o8b:A:199–218	1fjj:A	1lm4:A	1pcf:A
1d5t:A:292–388	1oew:A	1fmt:A:207–314	1m6y:A:115–215	1qqq:A
1sei:A	2aop:149–345	1a8d:248–452	1bkf	1kwf:A
1uro:A	4uag:A:1–93	1b8z:A	1chm:A:2–156	1dl5:A:214–317

**Table 12:** Validation dataset of 95 proteins

**Validation Dataset : Protein {:Chain}{:Domain Boundaries}**			
1d8h:A	1jfl:A:1–115	1bgf	1nxu:A
1dce:A:242–350	1jhg:A	1eu1:A:626–780	1o04:A
1di2:A	1ji7:A	1euw:A	1o6v:A:33–416
1dtj:A	1k2y:X:5–154	1f4l:A:389–548	1obo:A
1e0t:A:70–167	1klx:A	1f5n:A:284–583	1on2:A:63–136
1e85:A	1l3k:A:8–91	1f7l:A	1opd
1e8c:A:3–103	1lb6:A	1goi:A:447–498	1qh5:A
1eaq:A	1lc5:A	1gwy:A	1qlm:A
1ef1:C	1mrj	1hw1:A:79–230	1sox:A:94–343
1ewf:A:1–217	1o0w:A:-1–167	1hw5:A:1–137	1zfj:A:95–158
1f0j:A	1o1z:A	1i40:A	2mhr
1feh:A:210–574	1o6s:B	1i4m:A	2pvb:A
1fyf:A:242–532	1ogw:A	1j09:A:306–468	3sil
1g6s:A	1oi1:A:33–135	1jz8:A:731–1023	1aol
1g8t:A	1qcs:A:86–201	1k7i:A:259–479	1b6a:110–374
1gz8:A	1qnt:A:6–91	1kmv:A	1by2
1ifr:A	1t1d:A	1ku3:A	1c96:A:529–754
1ijq:A:377–642	1wpo:A	1l3p:A	1dhn
1iq4:A	1a12:A	1lfw:A:187–382	1dlj:A:295–402
1iqy:A:9–96	1a3a:A	1lsh:A:285–620	1dqa:A:587–703
1itx:A:338–409	1aop:81–145	1m1h:A:51–131	1dzf:A:5–143
1iu8:A	1b8o:A	1nm2:A:134–195	1e2w:A:1–168
1iwl:A	1bdo	1nte:A	1ekj:A
1jcl:A	1bfd:2–181	1nwa:A	

### Simulating negative training examples

The 285 native structures in Table 11 form the set of positive training examples. Negative training examples are derived by creating simulated decoy structures. This is done by reversing the sequence of each of the 285 native protein structures, and then threading the reversed sequence back onto the structure. This sequence reversal method is a reasonable first approximation to near-native decoys compared to structures with purely random sequences because some information regarding sequence order and neighbourhood composition of residues is retained in the reversed sequence. Here it should be pointed out that even though certain protein domains in the training dataset have domain boundaries (as shown in Table 11) and do not span the entire polypeptide chain, the reversal of sequence is done on the entire polypeptide chain, and the final 'reversed' decoy structure is then extracted using the original domain boundaries. This is to ensure that the original chunk of 3D structure is retained, with only the sequence modified.

As discussed earlier in the Results section, the sequence reversal method of generating a decoy structure retains native structural features. This ensures that we do not have to contend with steric clashes or impossible large distances from non-protein like features when producing negative training examples. In the set of negative training examples, the reversing of the sequence will cause changes to the distributions of sequence-related features such as the pairwise distance. The idea of such a representation is to be tested in this paper, in conjunction to the usage of neural networks, to see if it is effective in discriminating near-native decoys.

Here two types of testing are performed. The first type is a preliminary test, where random decoy structures are created from the test dataset of 95 protein domains. This is done by randomly shuffling the residues in each native structure in the preliminary test dataset in Table 5. For each native protein in Table 5, 50 random decoy structures are generated. Preliminary testing is simply done to test the viability of the proposed machine learning paradigm for decoy discrimination and to decide upon a reasonable set of input features to allow the neural networks to separate native folds from random decoy structures.

The main benchmark testing on the final neural networks is where challenging 'real' decoys are generated by fragment assembly methods. This is discussed in the next section.

### Decoy datasets for testing

Decoy discrimination methods require decoy datasets for testing. In this work, the Baker decoy dataset[16] and the Decoys 'R' Us [28] suite of decoy datasets are used for testing the effectiveness of the proposed decoy discrimination method. In the Baker dataset[16], only the X-ray structures are selected.

Table 2 shows the decoy datasets, and the number of proteins for each dataset. Each decoy dataset consists of several native protein structures and the corresponding set of decoy structures for each native structure. The number of proteins listed in Table 2 may not correspond to those in the literature because some proteins in the datasets are already obsolete.

The following equation shows the Z score which is used to measure the extent of which a decoy discrimination method can select the native structure from among the decoys.

$$Z=\frac{S_{native}-\hat{S}}{\sigma_{S}}$$

where *S*_*native *_is the score of the native structure produced by the proposed decoy discrimination method, $\hat{S}$ is the mean score of all decoy structures, including the native structure, and σ_*S *_is the standard deviation.

For each protein in a decoy dataset, there exists a set of decoy structures with varying RMSDs. In a CASP scenario, the native structure is unknown to predictors. Therefore, the Z score can only be calculated in the aftermath of CASP for the native structure. Hence another performance measure is required for the benchmarking of the proposed decoy discrimination method.

Such a performance measure exists in the form of the enrichment score, introduced by David Baker and co-workers[16]. The aim of this measure is to quantify the degree of which the decoy discrimination method succeeds in identifying the lowest RMSD near-native structures. The enrichment factor or score[16] is the proportion of low RMSD decoys in a low energy subset of the decoy population, over the total number of low RMSD decoys in the entire decoy population. In the current context, the term 'low energy' would be replaced by 'high score'.

To quantify this, David Baker and co-workers use 15% as the thresholds for the cut-off for both the low RMSD decoy subset and the low energy subset. In the following equation, the enrichment score[16] is defined as the intersections of both the subsets divided by what might be expected for a uniform distribution of low-energy decoys as well as for low RMSD decoys. Values greater than one suggest that the decoy discrimination method has an enrichment over a uniform distribution [16],

$$enrichment=\frac{M_{15\%}\cap R_{15\%}}{15\%\times 15\%\times N}$$

where *M*_15% _is the list of decoys with the top 15% highest scores as identified by the decoy discrimination method, *R*_15% _is the list of decoys with the top 15% of lowest RMSDs, and *N *is the total number of decoy models.

For a decoy discrimination method, the ability to select a decoy model of good quality as its top ranked model is assessed using different structural similarity measures, namely TM-score[19], GDT-TS[20] and MaxSub[21]. Between two decoy discrimination methods, the one-tailed Wilcoxon sign-rank test is used to test, at a 5% significance level, the null hypothesis of zero median in the distribution of differences in structural similarity scores of the respective top ranked models produced by the two methods. The one-tailed Wilcoxon sign-rank test can be repeated for the assessment of the difference, if any, in the distributions of any two decoy discrimination methods in terms of the overall Spearman rank correlation coefficients of both methods. The Spearman rank correlation coefficient of a method measures the correlation between the output scores of each method and the structural similarity (e.g. TM-score [19]) score of the decoy structures.

### Neural network input features

In this work, the strategy of using neural networks for decoy discrimination, using native structures as positive training examples and simulated decoy structures as negative training examples, is to present to the networks sufficient examples of features of correct and erroneous protein structures in the hope that they can learn to pick out native or near-native structures, based on these features, from a set of decoys.

With a set of positive and negative training examples comprising of native protein structures and simulated decoy structures, input features are obtained from each structure. The input features are the identities of all possible residue pairs along a sequence, with a particular sequence separation *k*, the pairwise distance (in Å) between the residues, and the relative solvent accessibilities of these residues. Residue pairs with sequence separation *k *= 3 are excluded.

The pairwise distance between residues is defined as the distance between corresponding Cβ atoms. Virtual Cβ atoms are created for Glycine residues. For non-Glycine residues occupying Glycine positions after the reversal of sequence, the virtual Cβ atom positions are also used.

To obtain the relative solvent accessibilities of the residues, the absolute solvent accessibility values of the residues are first obtained from the DSSP program[29] and normalized by dividing each absolute solvent accessibility value by the maximum solvent accessibility value of that residue[30]. Solvent accessibility values of the residues in the simulated decoy structures with reversed sequences are also obtained using DSSP [29], and normalized accordingly.

For example, a typical input feature vector is (Ala, Ala, *k *= 6, 11.4Å, 0.35, 0.41) where the Alanine pair is separated by 6 residues apart in the sequence, and whose Cβ atoms are 11.4Å apart in the structure, and the first and second Alanine residues have relative solvent accessibilities of 0.35 and 0.41 respectively.

In the training data, input features extracted from native structures are given the labels of '1', while input features extracted from simulated decoy structures are given the labels of '0'.

Due to the lack of computational resources of CPU power and memory required to handle one single large set of training data, the training data is divided into 20 sets, one set for each value of sequence separation *k *from 4 to 22 inclusive, and one set for *k *> 22. For the *k *> 22 set of training examples, due to its large size, 1 out of every 10 positive and negative training examples are selected from the positive and negative training data respectively. The validation data is also partitioned likewise.

Different network topologies are selected for different levels of inclusion of features. This is done to see the effects of the various input features on the decoy discrimination process. Therefore 20 neural networks are required for each instance of network topology.

### Neural network training

**Figure 8:**
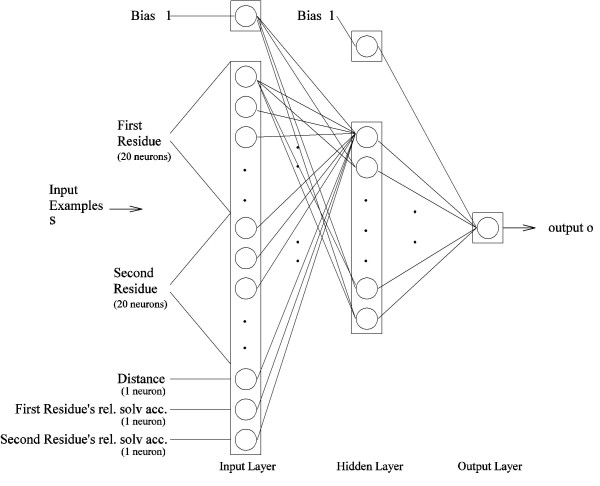
Neural network topology.

Figure 8 presents the neural network topology of a particular sequence separation *k*, which shows how all of these input features are encoded. For the identity of the residues, 20 input neurons are used for each residue, one for each type of residue. To indicate the presence of a residue, say Alanine, the neuron representing Alanine is set to the input value of 1, and all the other 19 neurons is set to 0. The pairwise distance uses 1 neuron, and the relative solvent accessibilities of the residues occupy 2 neurons.

Different sets of neural networks are designed to be trained on different levels of inclusion of the number of features. The different sets of neural network training paradigms are summarized in Table 3. The identities of the residue pairs are used in all training paradigms, as shown in Table 3. NN-dist includes only the pairwise distance; NN-solvpair includes only the relative solvent accessibilities of the residues; NN-solvpairndist includes all of the features. Figure 8 refers to the NN-solvpairndist method.

The batch method of neural network training is used. During training, the input training examples with output labels '1' or '0' are presented to the neural network of a particular sequence separation *k*, and the neural network would attempt to minimize the Mean Square Error (MSE) until the error on the validation dataset starts to increase or until the MSE falls below 0.01. Due to the nature of the training examples where positive and negative training data can have similar input vectors but differing output labels, each network is not expected to achieve low MSE.

The Matlab Neural Network toolbox is used for this work. A 2-layer feedforward neural network is used in all cases. For each sequence separation *k*, the number of hidden units is varied from 4 to 16 inclusive, and the topology with the number of hidden units that yields the lowest MSE is selected. Tests have shown that the radial basis function is the most suitable transfer function, and for the network training algorithm, the Levenberg-Macquardt algorithm yields the lowest MSE (data not shown).

The input features in the training dataset, with different levels of inclusion, are used for the training of the neural networks. The validation dataset is also preprocessed into these features for the use of early stopping in the neural networks. During the testing on the various decoy datasets, each set of decoy structures, together with the native structure, in the dataset is also preprocessed into these input features and fed into the appropriate neural network for testing.

### Interpretation of network output

During the testing phase, similar preprocessing is carried out on each of the decoy, and native, structures in a dataset. Each structure is broken into vectors of input features and fed into the appropriate neural network of sequence separation *k*.

A high network output score of a test vector would indicate that this vector is likely to come from a native structure, and vice versa. A test structure, native or decoy, would have outputs of several test vectors for a particular sequence separation *k*. For each structure with each sequence separation *k*, the outputs of the test vectors of that particular neural network of that sequence separation *k *are averaged to produce a single mean score. There is therefore a need to combine these single averaged network scores of different *k*.

**Figure 9:**
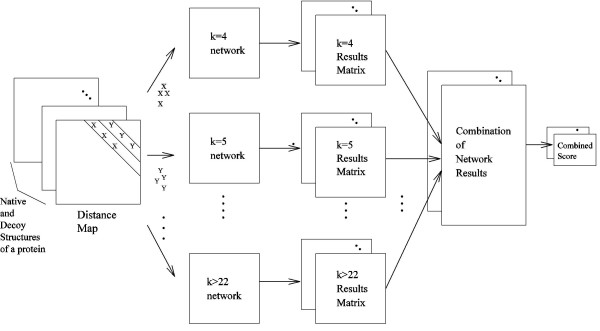
Neural network method of decoy discrimination.

Figure 9 shows how each structure is assigned a combined score from among the various neural networks. These average scores of each sequence separation *k *are then summed in 3 different ways. The short-range (S) combination sums up the average scores from the *k *= 4 to *k *= 10 networks; the short-and-medium (SM) combination sums up the average scores from the *k *= 4 to *k *= 22 networks; the short-medium-long (SML) combination sums up the average scores from the *k *= 4 to *k *= 22, as well as *k *> 22, networks.

The Z score and enrichment score[16] can then be calculated for the scores of the various structures, each of these types of combination.

### Methods for comparison

The in-house pairwise potentials of mean force method [17], and the K Nearest Neighbours algorithm, are used for comparison against the proposed decoy discrimination methods.

### Pairwise potentials of mean force

The pairwise potentials of mean force, used in mGenTHREADER[31] and FRAGFOLD [17], can also be used for decoy discrimination, as in the case of the MQAP method MODCHECK[9]. Here, it is used as a means of providing a benchmark for the proposed neural network method.

The following equation shows how the net potential of a residue pair *ab*, with sequence separation *k *and distance interval *s*, is calculated. The distance is taken between Cβ-Cβ atoms of the residue pair. In the case of Glycine, an approximate Cβ position is calculated.

$$\Delta E_{k}^{ab}=RT\ln[1+m_{ab}\sigma ]-RT\ln[1+m_{ab}\sigma \frac{f_{k}^{ab}(s)}{f_{k}(s)}]$$

The term *m*_*ab *_is the number of pairs *ab *observed with sequence separation *k*, σ is the weight given to each observation and is set to 0.02, *f*_*k*_*(s) *is the frequency of occurrence of all residue pairs at topological level *k *and separation distance *s*, *f*_*k*_^*ab*^*(s) *is the equivalent frequency of occurrence of residue pair *ab *and *RT *is taken to be 0.582 kcal/mol.

In the comparison of Z scores, the magnitude of the sign of the Z scores produced by the pairwise potentials method[17] are inverted in order to make effective comparisons with those produced by neural networks.

### K Nearest Neighbours algorithm

The K Nearest Neighbours (K-NN) algorithm is a common machine learning classifier that takes a particular test example and assigns to it the class where the majority of the K nearest training data points belongs to. The K-NN method can be used in the context of the proposed decoy discrimination method.

The training data used for classifying test data is the same as that used for training of the neural networks. For each separation *k*, there exist 400 sets of training data with both types of labels *'1's *and *'0'*s (for each type of residue pair) which the K-NN classifier can be applied to, depending on the particular test data point. For each decoy (and native) structure, there exists a set of test data points in the form of (*R1*, *R2*, *d*) for each separation *k*. Each of these test data points (with a particular distance *d*) is then used to select the K nearest neighbours in the training set of (*R1*, *R2*) where the training data points are of the class label *'1' *or *'0'*. The distance measure used to classify 'nearest' is that of the standard Euclidean distance.

The number of neighbours used in the benchmarking is 10 and 100. Instead of assigning an absolute *'1' *or *'0' *to the test data point, the ratio of the number of training data points with labels *'1'*s to the number of training data points with labels *'0'*s is taken.

### Inclusion of evolutionary information

In this work, we propose that the inclusion of evolutionary information can improve the decoy discrimination process, in the current context of the usage of neural networks. Evolutionary information in the form of multiple sequence alignments and derived profiles has been used successfully in several secondary structure prediction methods.

Two ways of including multiple sequence alignment information are proposed in this paper. The first way is to obtain the top 10 homologues of the target sequence identified using PSI-BLAST[1], and then thread these homologous sequences onto each structure in the decoy set, including the native structure. For example, if the *1hyp *protein of the Baker decoy dataset[16] has only 4 homologous sequences, these 4 sequences are threaded onto each of the decoy and native structures, scored using the trained neural networks, and the resulting scores averaged. This simple idea of averaging across homologues is widely used, for example by Finkelstein and co-workers in gapless threading[32]. The usual Z score and enrichment score[16] evaluations can then be applied to these mean scores.

**Figure 10:**
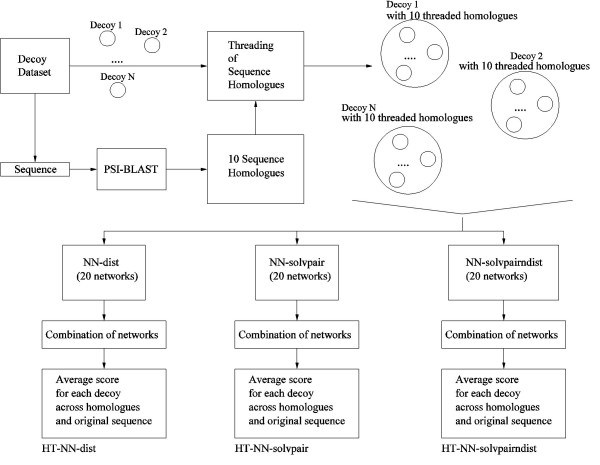
Homologue threading method.

For the sake of convenience, this particular way of using multiple sequence information is referred to as the homologue threading method. The motivation of the homologue threading method is primarily to reduce the noise of the neural-network based decoy discrimination method by applying it to many related sequences, instead of just one sequence, and then averaging the scores obtained. This is done under the assumption that the close homologues adopt similar 3D structures to that of the native sequence. The previous neural networks used for the homologue threading method are NN-dist, NN-solvpair and NN-solvpairndist shown in Table 4. Figure 10 shows an outline of the homologue threading method.

The second, and more rigorous, way to make use of evolutionary information is to develop a variant of the neural network method that is trained on sequence profiles, instead of just residue identities. The neural network topology is selected in such a way that there are 20 inputs per residue, and an additional 1 to 3 inputs depending on the feature of interest (pairwise distance and/or relative solvent accessibilities). The two 20 × 1 input vectors in Figure 8 are for single residue identities, with only 1 out of 20 neurons switched on for each training example during neural network training. Such an input topology is deliberately selected with the eventuality of training with evolutionary profiles in mind.

In this second method, the 20 × 1 input vectors would take in profiles of the residue positions, instead of the residue identities. These profiles are calculated from multiple sequence alignments of the original sequence. The input features of pairwise distance and/or relative solvent accessibilities are retained. This second method is labelled as the sequence profile method. There are 3 possible configurations of the sequence profile method, namely the topologies with the input feature of pairwise distance only, relative solvent accessibilities only, and a combination of both the pairwise distance and the relative solvent accessibilities.

For the sequence profile method, PSI-BLAST [1] profiles are generated for each sequence in the test decoy datasets. These PSI-BLAST[1] profiles are used directly as input to the neural networks, as in the case of the PSIPRED secondary structure prediction method[14]. A simple scaling formula, shown in the following equation, can be applied to each of the 20 values as part of the normalization process,

$$f(x)=\frac{1}{1+e^{-x}}$$

where *x *is the integer value obtained from the PSI-BLAST [1] Position Specific Score Matrix (PSSM).

For each sequence, 3 PSI-BLAST [1] iterations are run (-j 3). The parameters used in PSI-BLAST[1] are 0.001 for the initial and subsequent E-values thresholds (-h 0.001), and the sequence database used is UniRef50, release 6.7 [33]. These parameters are used for both the sequence profile methods and the homologue threading methods.

## Authors' contributions

CWT and DTJ worked on the design aspects of this method, and prepared the manuscript together. CWT did the implementation and carried out the testing.
